# Nutraceutical Use among Patients with Chronic Disease Attending Outpatient Clinics in a Tertiary Hospital

**DOI:** 10.1155/2020/9814815

**Published:** 2020-11-07

**Authors:** Nor Akila Mahmood, Mohd Rohaizat Hassan, Saharuddin Ahmad, Haniff Mohd Nawi, Nicholas Tze Ping Pang, Syed Sharizman Syed Abdul Rahim, Mohammad Saffree Jeffree

**Affiliations:** ^1^Department of Community Health, Faculty of Medicine, National University of Malaysia, 56000 Cheras, Kuala Lumpur, Malaysia; ^2^Department of Pharmacy, Raja Perempuan Zainab II Hospital, 15586 Kota Bharu, Kelantan, Malaysia; ^3^Department of Family Medicine, Faculty of Medicine, National University of Malaysia, 56000 Cheras, Kuala Lumpur, Malaysia; ^4^Department of Community and Family Medicine, Faculty of Medicine and Health Sciences, Universiti Malaysia Sabah, 88400 Kota Kinabalu, Sabah, Malaysia

## Abstract

Food-drug interphase products, referring to nutraceuticals in this study, are a category of health products containing a combination of food ingredients with active substances for oral consumption. Many of these products are marketed as an alternative to prescription medicine to treat various ailments despite the lack of scientific evidence, influencing patients with chronic diseases to consume nutraceutical products. This study aimed to determine the prevalence and factors associated with knowledge, attitude, and practice of nutraceutical use among patients with chronic disease attending to the outpatient clinic. This is a cross-sectional study involving patients with chronic disease at the outpatient specialist clinic. Samples were recruited from the outpatient clinic using convenience sampling. Data was collected using a self-administered questionnaire, which was self-constructed and validated. We found that the use of nutraceuticals was prevalent among 17.9% of respondents. More than half (60.9%) of the respondents have poor knowledge of nutraceutical and 53.1% of respondents have a positive attitude towards nutraceutical. Gender and morbidities were the factors associated with the practice of nutraceutical usage. Female patients are more likely to have increased use of nutraceutical than male patients and patients with multiple morbidities have higher odds of using nutraceutical than patients with single morbidities. There is a high number of patients who consume nutraceutical products and public knowledge of nutraceutical needs to be improved further. The government should develop appropriate regulation and monitoring of nutraceutical products.

## 1. Introduction

The advent of technological development and medical advancement has increased human life expectancy. Living longer, however, also exposes people to various chronic diseases. For example, the number of people worldwide diagnosed with diabetes mellitus keeps increasing from 151 million in 2000 to 425 million in 2017 [1]. Worryingly, the prevalence of diabetes mellitus is predicted to further increase by 48% in 2045. Apart from conventional medicine, various forms of complementary and alternative medicines (CAMs) are utilised to manage and treat these diseases. Some CAMs have a deep history and their efficacy has been proven over time, such as traditional Chinese medicine, Ayurvedic medicine, and acupuncture, whereas, some CAM like nutraceuticals have dubious effectiveness due to the lack of scientific evidence and proper research.

Nutraceutical is first introduced in 1989 by Dr. Stephen L. Defelice, the founder of the Foundation for Innovation in Medicine, and the term is a combination of the words nutrition and pharmaceutical [2]. The term is defined by Defelice in 1994 as “any substance that may be considered as food or part of a food and provides medical and health benefits, including prevention and treatment of disease. In Malaysia, the term nutraceutical is not widely used. Instead, products that inhabit the grey area between food and drug, are sold with the intention to maintain, prevent or even treat chronic diseases are classified under food-drug interphase (FDI) products [3]. FDI products are defined as “products for oral consumption containing a combination of food ingredients with active substances for oral consumption.”

Being convenient and easily available, more patients with chronic diseases are taking nutraceuticals as part of the management of their condition. A recent study revealed that 56.9% of Malaysian older adults in urban community-dwelling use at least one dietary supplement together with their prescription medicine [4]. The same study also revealed an increase in the use of dietary supplements among patients with multiple morbidities and that dietary supplements account for 30.7% of the total medication used. Dietary supplement use is found to be the highest among older adults who are diagnosed with oncologic disorders, followed by bone and joint disorders and gastrointestinal disorders. A total of 69.6% of the study population who are taking dietary supplements are also taking at least five other medications and a majority of these patients do not disclose their supplement use to their healthcare professionals. Another study investigating the use of CAM in patients with diabetes mellitus type 2 in an outpatient clinic in Malaysia also found a high prevalence of CAM use (62.5%) [5]. Herbal products are the most commonly used CAM and 58.0% of the respondents opted for CAM as they believe in the efficacy of CAM to achieve better control of diabetes. Respondents also agree that CAM is easily available, is affordable, and has better value for money. By and large, reasons for taking nutraceuticals can be classified into three main themes: to prevent illness or complications from the illness, to improve physiological and psychological conditions, and to treat illness [6].

Nutraceuticals in Malaysia are regulated either by the National Pharmaceutical Regulatory Division (NPRA) or Food Safety and Quality Division (FSQD). Products that are regulated by FSQD should adhere to the Food Act 1983 and should not make any health or medicinal claims. However, many of these products are being advertised as the cure-all solution for numerous diseases, influencing many patients with chronic diseases to consume these products, despite the lack of scientific evidence. In fact, one of the more prominent cosmetic tycoons in Malaysia is recently found guilty of promoting her product as the cure to burns and joint and knee pains [7]. Before long, the widespread use of these products will pose a problem to the healthcare system as patients may opt for these products to manage their medical conditions due to its availability. A study conducted among patients prescribed with antihypertensive agents revealed that CAM use is associated with reduced adherence to antihypertensive medications among female patients [8]. Although many studies have been published on the use of CAM and dietary supplements, studies on nutraceutical use are still lacking.

With no strict regulations in place to control the marketing of FDI products in Malaysia, more products are claiming to be a safer alternative to prevent and treat numerous chronic diseases. Quantifying the number of patients who are currently engaged in using nutraceuticals will give us a more accurate magnitude of this issue and may help in the development of the appropriate regulation and monitoring of nutraceutical products in Malaysia. Furthermore, patients' knowledge and attitude towards nutraceutical use need to be examined in order to tailor the educational needs and programs that can be developed to ensure the optimum management of medical conditions. Hence, this study aimed to determine factors associated with the practice of nutraceutical use among patients with chronic diseases in the Universiti Kebangsaan Malaysia Medical Centre (UKMMC).

## 2. Materials and Methods

This is a cross-sectional study involving patients with chronic disease receiving treatment in selected outpatient specialty clinics in Hospital Canselor Tuanku Muhriz (HCTM) and UKM Primer Clinic. The selected outpatient specialty clinics in HCTM were Medical Specialty Clinic 1 and 2, Nephrology and SLE (Systemic lupus erythematosus) Clinic, and Oncology and Radiotherapy Clinic. This study was approved by the Medical Research Ethical Committee, Universiti Kebangsaan Malaysia Medical Centre (FF-2018-229). A pilot study was conducted in August 2018, and data collection for the main study was conducted from September 3^rd^, 2018, to December 14^th^, 2018. Convenience sampling was employed in this study. Patients who fulfilled the inclusion and exclusion criteria were selected and interviewed. Up to 20 patients were selected daily. Using Pocock's sample size formula for comparison between two proportions, a total of 326 subjects were needed to determine the influence of education level on nutraceutical use. An additional 10% was added to prepare for dropout and incomplete data. The final sample size needed to conduct this study was 360 subjects. The inclusion criteria were adult patients who were 18 years old and above diagnosed with chronic diseases under follow-up of selected HCTM outpatient specialty clinics (Medical Specialty Clinic 1 and 2, Nephrology and SLE Clinic, and Oncology and Radiotherapy Clinic) and UKM Primer Clinic. The exclusion criteria were patients who cannot understand Malay, those not of Malaysian citizenship, those who did not consent to be interviewed, and those who were diagnosed with active psychiatric conditions and unable to give informed consent.

The main instrument used in this study was a set of questionnaires developed by the investigators based on past literature. Patients are supposed to complete the questionnaire with the guidance of the interviewer. The questionnaire developed for this study consists of four sections (Section A: Profile, Section B: Knowledge, Section C: Attitude, and Section D: Practice) to measure all variables. The questionnaire is six-page long and contained a total of 28 items excluding the respondent's profile. A pilot study was conducted to ensure the construct validity of the questionnaire after experts have ensured its content validity. The questionnaire was piloted in 92 patients with chronic diseases attending HCTM Surgical Clinic and Orthopaedic Clinic. Eligible respondents were approached and guided interview using the questionnaire was conducted after obtaining consent. The same interviewer who conducted the guided interview in the pilot study would be conducting the interviews in the main study. Apart from answering the questions, respondents were asked about their general opinion on the questionnaire to improve the questionnaire. The respondents who participated in the pilot study were not included in the main study. Factor analysis for construct validity was conducted for items with Likert-scale option. Two constructs were successfully identified: self-perceived health status and attitude. Items 10 and 11 in Section A were found to belong to one construct (self-perceived health status) and all items in Section C except item 6 were identified as another construct. Item 6 of Section C was found to be in another construct and thus was omitted from the final questionnaire.

Section B originally comprised seven questions. After analysis of difficulty index (*D*) and discrimination index (*R*) was conducted, question seven was omitted due to very low *D* and *R* value.  Table 1 summarises the *D* and *R* value for each item in Section B. According to Universiti Kebangsaan Malaysia categorisation of *D* and *R* values, *D* value of <30 was categorised as difficult, 30 to 70 as average, and >70 as easy, whereas *R* value of 0.01 to 0.15 has poor discrimination, 0.16 to 0.25 marginal discrimination, 0.26 to 0.35 good discrimination and questions with *R* > 0.35 excellent discrimination. Cronbach's *α* was obtained to ensure internal consistency reliability. In Section A, the two questions on self-perceived health status garner Cronbach's *α* value of 0.52, which was a little low. Both questions, however, were retained after discussion with an epidemiologist. In Section C which measures attitude, Cronbach's *α* value of 0.95 was obtained after the exclusion of item 6, indicating high reliability.

Approval from the Review Board and Ethics Committee UKM was obtained preceding the study. Prior to face-to-face interview, eligible patients were briefed on the purpose of the study based on the patient information sheet. Patients were assured that no personal details that can identify them were collected and all information was kept confidential and only used for research purposes. Patients who agreed to participate in the study were required to sign a written consent form before face-to-face interview was conducted. Upon the return of completed questionnaires, raw data was processed and entered into Statistical Package for Social Sciences (SPSS) version 23.0 for data analysis. For objectives 1 to 3, the descriptive analysis (frequency and percentage, mean and standard deviation) was carried out. The bivariate analysis was conducted (*t*-test, chi-square and Mann–Whitney *U* test) to determine the association between the independent variables on the use of nutraceutical. Also, multiple logistic regression was conducted to analyse for confounders.

## 3. Results

A total of 341 respondents from outpatient clinics of UKMMC completed the questionnaire handed in the study. A majority of respondents were recruited from the Medical Clinic (42.8%, *n* = 146), followed by Primer Clinic (30.8%, *n* = 105) and Oncology Clinic with 50 respondents (14.7%). Only 11.7% (*n* = 40) respondents were recruited from Nephrology Clinic. Slightly more than half (54.5%) of the respondents suffered from single morbidity, whereas 45.5% (*n* = 155) of respondents were diagnosed with at least two chronic diseases. Among the most commonly diagnosed diseases were diabetes mellitus (32.0%) and hypertension (32.0%). A summary of the demography of respondents is depicted in Table 2.

The mean score for knowledge was recorded to be 7.2 (±SD 1.6). Less than half of the respondents scored high marks in the knowledge section of the questionnaire, with only 39.3% (*n* = 134) achieving at least eight marks (Table 3). The highest mark scored was 11, achieved by 2.3% (*n* = 8) of respondents, whereas 8.8% (*n* = 30) of respondents scored the lowest mark of 5. The mean score for attitude was 26.7 (±SD 9.7), with the highest mark being 43 and the lowest mark being 10. Using mean as the cut-off point, 53.1% (*n* = 181) of the respondents regard nutraceutical use positively and 46.9% (*n* = 161) have a negative attitude towards nutraceutical. However, only 17.9% (*n* = 61) of the respondents in this study have ever used nutraceutical in the past 12 months, with 9.7% (*n* = 33) of them still consuming nutraceutical in the past 30 days. Of these respondents, 3.5% (*n* = 12) respondents were regular and current consumers of nutraceutical, as indicated by their practice score of 8. These regular consumers of nutraceutical consistently take their nutraceutical product daily for at least five days of the week for the past 12 months. Of the 61 nutraceutical users, 50.8% (*n* = 31) are from Medical Clinic, 24.6% (*n* = 15) are from Primer Clinic, 13.1% (*n* = 8) from Oncology Clinic, and 11.5% (*n* = 7) are from Nephrology Clinic.  Table 4 shows the chronic diseases diagnosed for the 61 nutraceutical users and the usage of nutraceutical products in 61 of the respondents is portrayed in Table 5.

Among the many sociodemographic factors measured, only gender and age were statistically significant (Table 6). Female is found to be associated with more use of nutraceutical products compared to male with *p* value of 0.03, whereas the mean age of nutraceutical user was found to be slightly higher than nonuser. Monthly household income was also associated with the practice of nutraceutical use. Mann–Whitney *U* test conducted on monthly household income results in *p* value of 0.030, giving a statistically significant difference in the median income of the two groups. The user of nutraceutical had a median monthly household income of RM5000 (4000, 6000), which was higher than the median income of nonuser with RM4000 (3000, 5000). Morbidities were associated with nutraceutical use; 23.2% of the nutraceutical users were inflicted with multiple morbidities compared to 13.4% of them suffering from single morbidity. The chi-square test conducted yields a *p* value of 0.019, indicating a statistically significant difference in the morbidity status of nutraceutical users.

Table 7 shows a summary of factors associated with nutraceutical use by multiple logistic regression. Females were 2.255 times more likely to use nutraceutical compared to male patients and patients diagnosed with multiple morbidities had 2.237 odds of using nutraceutical products than patients who afflicted with single morbidity. Age was not statistically significant, suggesting that age is a confounding factor. Comparing Wald values, morbidities (7.445) contributed slightly more to nutraceutical use than gender (6.513). No interactions between the two variables were found and the variance inflation factor (VIF) was less than 10, suggesting that multicollinearity was not a problem in this model. Hosmer–Lemeshow goodness-of-fit test was not significant (*p*=0.487), indicating that the model fits well with our findings and the model was able to predict 82.1% of cases correctly. Unfortunately, Nagelkerke R square of 0.059 indicates that this model only accounts for 5.9% of factors associated with nutraceutical use.

## 4. Discussion

The prevalence of nutraceutical usage among patients with chronic disease attending outpatient clinics of Universiti Kebangsaan Malaysia Medical Centre is found to be 17.9%. Compared to other studies investigating the prevalence of other CAM use in Malaysia, our finding is considerably smaller. Hasan et al. [9] reported that 63.9% of patients attending outpatient clinics of a general hospital in Malaysia use CAM concurrently with their prescription medicine. However, it should be noted that this study examined the prevalence of CAM use in general and do not limit to one form of CAM. Compared to our study that focuses only on food-drug interphase products, other studies include vitamins, dietary supplements, herbal medicine, and various other CAMs. Thus, it is reasonable for our findings to have a lower prevalence than other studies investigating CAM use in Malaysia.

Another reason attributed to the smaller percentage of nutraceutical users is that nutraceutical products are not as widely spread as other CAM products, such as vitamins and dietary supplements and herbal medicines. The concept of nutraceutical, although not new, is more recent than other CAMs. Moreover, it is known that CAM use is transient in nature. As we only measure nutraceutical usage in the 12 months prior to the study period, it is not known if there are respondents in this study who has ever consumed nutraceutical in a longer period. In this study, only 9.7% (*n* = 33) of respondents are currently taking nutraceuticals, whereas the remaining nutraceutical users are no longer consuming nutraceuticals in the previous 30 days, hence giving rise to a smaller percentage of patients taking nutraceutical products.

From this study, most of the nutraceutical users consume nutraceutical products to treat or prevent complications of diseases and to maintain general well-being. This finding is in concordance with the reasons for taking CAMs proposed by Pajor et al. [6]. Rather than using nutraceutical to treat the disease that they are diagnosed with, similar to the findings reported by Djuv et al. [10], where most of the respondents use nutraceutical to alleviate the complications of their disease and improve their physiological conditions, Farooqui et al. [11] also found similar results among cancer patients who use CAMs. This is also supported by the fact that all 61 of nutraceutical users continue to take their prescribed medicine despite incorporating nutraceutical in the management of their disease. Hasan et al. reported in both of their studies that patients who have family members who use CAMs are more likely to take up CAM. Unfortunately, we are unable to ascertain this suggestion due to the lack of data, but most of the nutraceutical users in our study obtained information on nutraceutical products from their family members.

Contrary to Egan et al. [12] and Samuels et al. [13], who found commendations from healthcare professionals to play important roles in CAM use among patients with chronic disease, none of the respondents in this study seek information about nutraceutical from healthcare staffs. This is perhaps due to the fact that there is lacking evidence on the safety and effectiveness of nutraceutical. Thus, healthcare staff do not have the confidence to recommend nutraceutical products to their patients. Also, 60.7% (*n* = 37) of the nutraceutical users do not inform their healthcare providers of their nutraceutical use, a finding that is common among patients in Malaysia and Thailand [9, 14–16]. Because they do not see the need to disclose information on their nutraceutical use, healthcare providers may not be aware of their nutraceutical use and unable to make recommendations in regard to nutraceutical products.

Section B of the questionnaire aims to measure respondents' knowledge of food drug interphase products, referred to as nutraceutical in this study. None of the respondents managed to achieve full mark and only 39.3% of respondents are found to have good knowledge of food drug interphase products. Compared to Kumar et al. [17] who reported 71% of diabetic patients with high awareness of CAM, the respondents in our study lack awareness of nutraceutical. Furthermore, most of the respondents in this study are not fully knowledgeable about the division of nutraceutical regulation between National Pharmaceutical Regulatory Division (NPRA) and Food Safety and Quality Division (FSQD), as evidenced by only 38.4% of respondents who correctly answered the question on the governance of FSQD over nutraceutical. This is much lower than what is reported by Owens et al. [18], where 61.1% of the rural population in South Idaho were aware of the role of FDA in the regulation of dietary supplements. Hence, it can be deduced that there is a lack of correct information on food-drug interphase products. In fact, almost none of the respondents are aware of the term food-drug interphase products nor nutraceutical at the beginning of the study. Only after explanation from the investigator did the respondents understand what nutraceutical refers to.

In terms of attitude towards nutraceutical use, the proportion of respondents who sees nutraceutical positively is similar to those who have negative views on nutraceutical. This is perhaps because respondents are used to the idea of complementary and alternative medicine as Malaysia is rich with various forms of CAMs. Many studies have suggested the use of CAM among patients in Malaysia had reached up to 60% of the population [4, 5, 9]. Hence, it is reasonable to assume that the participants in this study have been exposed to at least one type of CAM in their life, shaping their view on nutraceutical as well. Furthermore, nutraceutical products are usually marketed to be safe and natural despite lacking in scientific evidence; thus, less prejudice is attached to these products.

Various sociodemographic parameters were examined in this study, but not all parameters were statistically significant in the bivariate and multivariate analysis. Female patients are linked with more use of nutraceutical products than male patients in this study. Many studies have also suggested that females have increased the use of CAM compared to males [5, 9, 11, 19–23]. Gender remains statistically significant after multiple logistic regression was conducted to adjust for confounders. Compared to Ching et al. [5], who reported that females have 1.8 more odds of using CAM compared to males, we found the odds ratio of females using nutraceutical to be slightly higher crude OR (1.9) and adjusted OR (2.3). Among the 12 current and regular users of nutraceutical, 10 are female, indicating that women are more likely to use nutraceutical consistently [24]. The female gender has always been said to be more health-conscious than male and hence is associated with higher health-seeking behaviours such as taking CAMs to improve their health and medical condition. This behaviour is also apparent in nutraceutical consumption as gender is found to be one of the factors associated with nutraceutical use in this study.

Other than gender, morbidity status is found to be associated with the practice of nutraceutical use. Lim et al. [4] and Falci et al. [25] reported that the frequency of CAM use is higher among adults diagnosed with two or more diseases. Multiple morbidities are a predictor of CAM use, even after adjustments for confounders and collinearity [25, 26]. Our study consolidates this finding as patients with multiple morbidities have crude OR (1.95) and adjusted OR (2.24) of taking nutraceutical products compared to patients diagnosed with single morbidity. Patients with multiple morbidities are likely to use more nutraceutical as they seek to manage various ailments and are more willing to try alternative options in treating and preventing complications of their diseases.

The results of this study are impeded by several limitations. Firstly, convenience sampling was employed for this study. Thus, the results obtained cannot be an inference to the whole population. Another limitation of the study is that the number of respondents from each clinic is not stratified according to the total number of patients attending each clinic. Therefore, the sample may not represent the population of patients attending outpatient clinics in Universiti Kebangsaan Malaysia Medical Centre. Although we tried to include patients of different demography, voluntary participation indicates a certain pattern of respondents. Older patients, for instance, are less likely to be included in the study. The prevalence of nutraceutical use in this study may not represent the whole population of Malaysia as the composition of respondents in this study is focused on patients attending Universiti Kebangsaan Malaysia Medical Centre, which is located in the urban areas. Extending this study to several other areas in semiurban and rural areas might produce different results. The subject of this study could be one of the limitations. FDI products are not widely known compared to other CAMs such as dietary supplements, vitamins, and herbal products. The term FDI and nutraceutical are new to most of the respondents. Thus, investigators had to explain what nutraceutical refers to during the recruitment of study participants. The lack of research on products such as FDI also limits our literature review, and we can only compare our findings to studies of other CAMs.

Based on our findings, it can be implied that there is a lack of accurate information on FDI products despite its use among patients with chronic diseases. Like other pharmaceutical products, accurate information and precautions should be made known to consumers to help them to make decisions regarding nutraceutical consumption. Patients with comorbidities are more likely to use nutraceutical, thus, increasing the risk of polypharmacy and nonadherence to prescribed medicine. Physicians and medical professionals should, therefore, be more intrusive of their patients' use of CAM including nutraceuticals and their adherence to prescribed medicine. It is also important for researchers to put more effort into establishing the safety and efficacy of nutraceutical so that consumers can have peace of mind in taking nutraceutical. If it is indeed advantageous for the management of patients' diseases and well-being, nutraceutical products should be marketed openly albeit under stricter regulation.

## Figures and Tables

**Table 1:** Difficulty and discrimination index for questions in Section B.

Question	Difficulty index (*D*)	Discrimination index (*R*)
1	45.65	1.00
2	19.57	0.72
3	67.39	1.00
4	21.74	0.80
5	39.13	1.00
6	26.09	0.96
7	2.17	0.08

**Table 2:** Sociodemographic profile of respondents.

Variable	*n* (*N* = 341)	%	Mean (±SD)
Gender			
Male	137	40.2	
Female	204	59.8	
Age (in years)			44.4 (±12.4)
Race			
Malay	226	67.1	
Chinese	83	24.6	
India	20	5.9	
Other	8	2.4	
Marital status			
Single	128	37.9	
Married	182	53.8	
Divorced	18	5.3	
Widowed	10	3.0	
Education			
No formal education	6	1.8	
Primary school	9	2.6	
Secondary school	119	35.1	
Diploma/certificate	155	45.7	
Degree	47	13.9	
Postgraduate degree	3	0.9	
Work sector			
Government	129	38.6	
Private	56	16.8	
Self-employed	50	15.0	
Not working	15	4.5	
Pensioner	44	13.2	
Housewife	22	6.5	
Student	18	5.4	
Income in RM^*∗*^ (*n* = 80)			4000 (3000, 5000)^*∗*^
Morbidities			
Single morbidities	186	54.5	
Multiple morbidities	155	45.5	
Duration of disease in years^*∗*^ (*n* = 118)			4.0 (2, 8)^*∗*^
Self-perceived health status			
Poor	25	7.3	
Good	316	92.7	

^*∗*^median, IQR.

**Table 3:** Knowledge, attitude, and practice regarding nutraceutical use.

Variable	*n* (*N* = 341)	%
Knowledge		
Good (≥8)	134	39.3
Poor (<8)	207	60.7
Attitude		
Positive (≥27)	181	53.1
Negative (<27)	160	46.9
Practice		
User (≥1)	61	17.9
Nonuser (=0)	280	82.1

**Table 4:** Chronic diseases diagnosed among nutraceutical users.

Chronic disease	*n* (*N* = 61)
Hypertension	27
Diabetes mellitus	21
Dyslipidaemia	15
Cancer	8
Chronic kidney disease	7
Stroke	4
Heart failure	3
Gout	3
Ischaemic heart disease	2
Osteoarthritis/osteoporosis	2
Chronic obstructive pulmonary disease	2
Others	6

**Table 5:** Nutraceutical usage among patients with chronic disease.

Practice	*n* (*N* = 61)	%
Reasons for nutraceutical use		
Prevent disease	9	14.8
Treat or prevent complication of disease	24	39.3
Maintain general well-being	18	29.5
Complements conventional medicine	0	0.0
Worried about side effect of conventional medicine	0	0.0
Belief in the efficacy of nutraceutical products	10	16.4
Do you take prescription medicine with nutraceutical products?		
Yes	61	
No	0	100.0
How do you take nutraceutical products with your prescription medicine?		
Right after or before taking medicine	9	14.8
Space time between nutraceutical and medicine by at least 4 hours	52	85.2
Other family members using nutraceutical		
Yes	17	27.9
No	44	72.1
Where do you get information about nutraceutical products?		
Family members	24	39.3
Friend/neighbours	12	19.7
Television/radio/magazine/newspapers	9	14.8
Internet	16	26.2
Where do you get your supply of nutraceutical products?		
Health shop/agent/retailer	45	73.8
Internet	16	26.2
Do you tell your healthcare professionals (Doctors/Pharmacists) about your nutraceutical use?		
Yes	24	39.3
No	37	60.7

**Table 6:** Bivariate analysis of factors associated with nutraceutical use.

Variable	Total *n* (%)	User *n* (%)	Non-user *n* (%)	x^2^ (df) or t	*p* value
Gender					
Male	137 (40.2)	17 (12.4)	120 (87.6)	4.682 (1)	0.030
Female	204 (59.8)	44 (21.6)	160 (78.4)		
Age in years (mean ± sd)	44.4 (±12.4)	47.4 (±11.1)	43.8 (±12.7)	2.053^#^	0.040^#^
Race					
Malay	226 (67.0)	42 (18.6)	184 (81.4)	0.285 (1)	0.593
Non-Malay	111 (33.0)	18 (16.2)	93 (83.8)		
Marital status					
Married	174 (51.0)	33 (19.0)	141 (81.0)	0.281 (1)	0.596
Unmarried	167 (49.0)	28 (16.8)	139 (83.2)		
Education					
Secondary and less	134 (39.5)	21 (15.7)	113 (84.3)	0.463 (1)	0.496
Tertiary	205 (60.5)	38 (18.5)	167 (81.5)		
Work					
Employed	235 (70.4)	44 (18.7)	191 (81.3)	0.112 (1)	0.737
Unemployed	99 (29.6)	17 (17.2)	82 (82.8)		
Income (median, IQR) (*n* = 80)	4000 (3000, 5000)	5000 (4000, 6000)	4000 (3000, 5000)	355.500^##^	0.030^##^
Morbidities					
Single morbidities	186 (54.5)	25 (13.4)	161 (86.6)	5.511 (1)	0.019
Multiple morbidities	155 (45.5)	36 (23.2)	119 (76.8)		
Duration of disease in years (median, IQR) (*n* = 118)	4 (2.0, 8.0)	4 (2.0, 8.0)	4 (2.0, 8.0)	1145.000^##^	0.907^##^
Self-perceived health status					
Good	316 (92.7)	57 (18.0)	259 (82.0)	<0.001^*∗*^ (1)	1.000^*∗*^
Poor	25 (7.3)	4 (16.0)	21 (84.0)		
Knowledge					
Good	134 (39.3)	25 (18.7)	109 (81.3)	0.089 (1)	0.766
Poor	207 (60.7)	36 (17.4)	171 (82.6)		
Attitude					
Positive	181 (53.1)	33 (18.2)	148 (81.8)	0.031 (1)	0.860
Negative	160 (46.9)	28 (17.5)	132 (82.5)		

^#^Student's *t*-test, ^##^Mann–Whitney *U* test; ^*∗*^Yates correction.

**Table 7:** Factors associated with nutraceutical use (using multiple logistic regression).

Variable	Crude OR (95% CI)	Adjusted OR (95% CI)	x^2^ (df)	*p* value
Gender	1.941 (1.057, 3.564)	2.255 (1.208, 4.212)	4.682 (1)	0.011
Age in years^*∗*^	1.024 (1.001, 1.048)	1.015 (0.987, 1.044)	2.053	0.298
Race	1.179 (0.643, 2.162)		0.285 (1)	0.594
Marital status	1.162 (0.667, 2.025)		0.281 (1)	0.597
Education	1.224 (0.683, 2.196)		0.463 (1)	0.497
Work	1.111 (0.600, 2. 059)		0.112 (1)	0.738
Morbidities	1.948 (1.110, 3.420)	2.237 (1.255, 3.990)	5.511 (1)	0.006
Self-perceived health status	1.155 (0.382, 3.496)		<0.001 (1)	0.798
Knowledge	1.089 (0.620, 1.195)		0.089 (1)	0.766
Attitude	1.051 (0.603, 1.832)		0.031 (1)	0.860

^*∗*^Age found to be confounder in adjusted analysis.

## Data Availability

The data used to support the findings of this study are available upon request to the authors.
